# Sugarcane Smut: Current Knowledge and the Way Forward for Management

**DOI:** 10.3390/jof7121095

**Published:** 2021-12-19

**Authors:** Muhammad Aslam Rajput, Nasir Ahmed Rajput, Rehana Naz Syed, Abdul Mubeen Lodhi, Youxiong Que

**Affiliations:** 1 National Sugar and Tropical Horticulture Research Institute, PARC, Thatta 43130, Pakistan; rajputaslam70@gmail.com; 2 Department of Plant Protection, Faculty of Crop Protection, Sindh Agriculture University, Tandojam 70060, Pakistan; rnsyed@sau.edu.pk; 3 Department of Plant Pathology, University of Agriculture, Faisalabad 38000, Pakistan; nasirrajput81@gmail.com; 4 Key Laboratory of Sugarcane Biology and Genetic Breeding, Ministry of Agriculture and Rural Affairs, Fujian Agriculture and Forestry University, Fuzhou 350002, China

**Keywords:** sugarcane, *Sporisorium scitamineum*, management, molecular markers, diagnostic, germplasm screening

## Abstract

Whip smut of sugarcane is the most serious and widely spread disease of sugarcane and causes a significant reduction in cane quantity and quality. The severity of this disease often depends on the pathogen races, environmental conditions, cultivar genotype and the interaction among these three factors. Under optimum climatic conditions, this disease has the potential to cause total crop failure. Resistance screening is an ongoing process due to the variability among smut pathogen isolates. Multiple races and mutation ability of smut pathogen makes the breeding task more complex. A number of studies on various aspects of the disease epidemiology and management have been published. Due to many overlapping characteristics within the species complex, there is a dearth of information on early detection and strategies to control the smut pathogen. Furthermore, there is a need to coordinate these findings to expedite its research and control. In this paper, we summarize the disease etiology, especially disease impact on the qualitative and quantitative parameters of sugarcane. We also gathered research progress on molecular-based detection and available information on genetic variability in *S.* *scitamineum.* The research on the set of management options needed to effectively cope with the disease are reviewed herein. The present review is expected to be helpful for the further investigation on smut resistance in sugarcane.

## 1. Introduction

**Figure 1:**
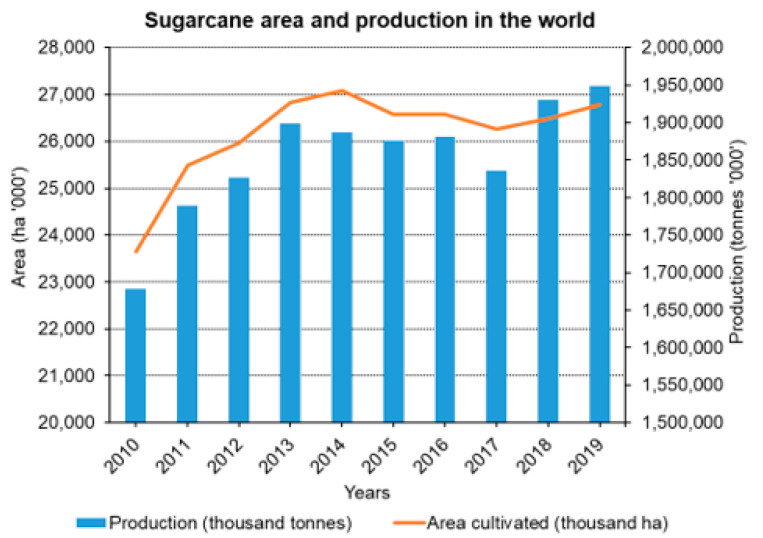
Worldwide sugarcane acreage and production from 2010 to 2019 (FAOSTAT, 2021).

Sugarcane, *Saccharum officinarum* L., is the world’s largest commercial crop, which is cultivated in more than 120 countries on about 26.27 million hectares, with a worldwide harvest of 1.90 billion tonnes (Figure 1). Approximately 80% of the world sugar is derived from sugarcane [1]. Harvesting of sugarcane provides hundreds of tons of green matters per hectare each year, and provides 75,000 million calories each year [2]. The sugarcane crop requires 12–14 months for maturity and harvesting, and during the long duration crop, it suffers from many biotic and abiotic factors. Individually, pathogens and insect pests have a potential to decrease its production up to 20% [3,4,5]. Among biotic agents, fungal pathogens are most challenging. More than 100 fungi have been reported to cause diseases in sugarcane [6]. The most considerable sugarcane diseases are whip smut, red rot, leaf blast, sugarcane mosaic virus, pineapple disease, ratoon stunting disease, leaf scald, mottled stripe, pokkah boeng and wilt [7,8].

The whip smut caused by *Sporisorium scitamineum* (Phylum: Basidiomycota, Order: Ustilaginales) is considered as the most serious and widely spread disease of sugarcane, known to affect both qualitative and quantitative components causing the substantial economic losses [9,10,11,12,13,14,15]. The severity of the disease often depends on the pathogen races, environmental conditions and cultivars grown [16,17]. It becomes more serious under favorable conditions, which can even cause a complete crop failure in extreme cases [18]. Besides the direct yield loss, the whip smut can cause the significant reduction in sucrose content, purity and other juice quality indicators [19,20,21]. Different races of *S. scitamineum* are known to exist. Random amplification of polymorphic DNA (RAPD), sequence-related amplified polymorphism (SRAP) [22], amplified fragment length polymorphism (AFLP) [23] and internal transcribed spacer (ITS) sequence analysis [24,25] have been applied to evaluate the intraspecific diversity among the *S. scitamineum* populations.

Microscopic examination for the presence of teliospores of *S. scitamineum* is a key identification parameter for sugarcane smut incursion [26]. By using species-specific primers, pathogens may be detected even earlier than the symptoms’ expression. Many workers successfully amplified and visualized *S. scitamineum* DNA present in the plant tissues by using *Ustilago maydis* primers bE4 and bE8 [27,28,29]. Moreover, the loop-mediated isothermal amplification (LAMP) method, which targets the core effector *Pep1* gene of *S. scitamineum*, has also been developed for the sensitive and rapid detection of smut pathogen [30]. Genome sequencing of *S. scitamineum* and its biotropic interaction transcriptome with sugarcane provides novel insights into the pathogenic mechanisms of sugarcane smut [31,32,33]. However, to measure the effects of disease on plant growth and yield, conventional screening methods are desirable and have been optimized by various workers [34,35,36].

Different measures are applied for control of sugarcane smut [34], such as hot water treatment, roughing out diseased plants, planting resistant or tolerant cultivars and the application of fungicides [4,8,37,38,39,40,41,42]. Fungicides not only eradicate smut from the planting material, but also defend seed cane from infection of pathogen inoculum present in the planting soil [43]. However, the application of fungicides in sugarcane (sett treatment) is not only laborious and costly, but also possesses harms to eco-system. High economic importance of the disease implies a stringent need of development for the effective integrated smut management programs [44]. Reliable, cost effective and eco-friendly disease control can only be achieved through resistant varieties. Cultivation of resistant varieties is the only reliable and practicable control measure to minimize the adverse impact of whip smut disease. Therefore, screening of sugarcane germplasm for smut resistance and other desirable characters is an ongoing process. In the past, several superior varieties have been eliminated for cultivation due to this disease [45,46,47]. Existing cultivated sugarcane cultivars are polyploidy or aneuploid, because they are originated from the interspecific hybridization between the *Saccharum officinarum* and its wild relatives i.e., *S. spontaneum* L., *S. sinense* Roxb. or *S. barberi* Jesw [12,28,48]. The resulting aneuploid progenies get stalk and high sucrose characters from *S. officinarum* (2n = 80, x = 10) and other desirable characters especially biotic and abiotic resistance from wild species of genus *S. spontaneum* (2n = 40–130, x = 8).

Breeding of new sugarcane varieties is difficult, complicated and time-consuming [49,50,51]. Traditionally, conventional breeding practices have comprised the development of insect pest and disease resistant varieties along with prerequisite yield and qualitative characters for a long time, but these procedures are particular costly and taking prolong periods for the development of a new cultivar. In addition, phenotypes correlated with many traits of attention are limited and often suffered by many environmental factors that make their identification more difficult and unpredictable, and on the basis of phenotype, the genetic potential of germplasm most probably cannot be estimated properly [52,53,54]. The introduction of molecular markers and their application in the field of agriculture enable the plant breeders to investigate genetic diversity of plant materials at the DNA level [55,56]. Differentially expressed genes and enzymes as a response to sugarcane–smut pathogen interaction and responsive gene related to sugarcane resistance are identified using cDNA-AFLP, cDNA-SRAP and suppression subtractive hybridization (SSH) [57,58,59,60].

Previous investigations have revealed that polygenic resistance towards this disease is present in sugarcane cultivars, as suffered plants show different types of reaction [61]. Sugarcane plant genes resistant to whip smut pathogen have been tagged [62]. It was also demonstrated that genetic diversity exists within the whip smut pathogen population in many parts of the world that need to be monitored [22,63]. This review presents current knowledge on the various aspects of sugarcane smut, and more importantly, recommends research directions for better disease management.

## 2. Disease Biology

**Figure 2:**
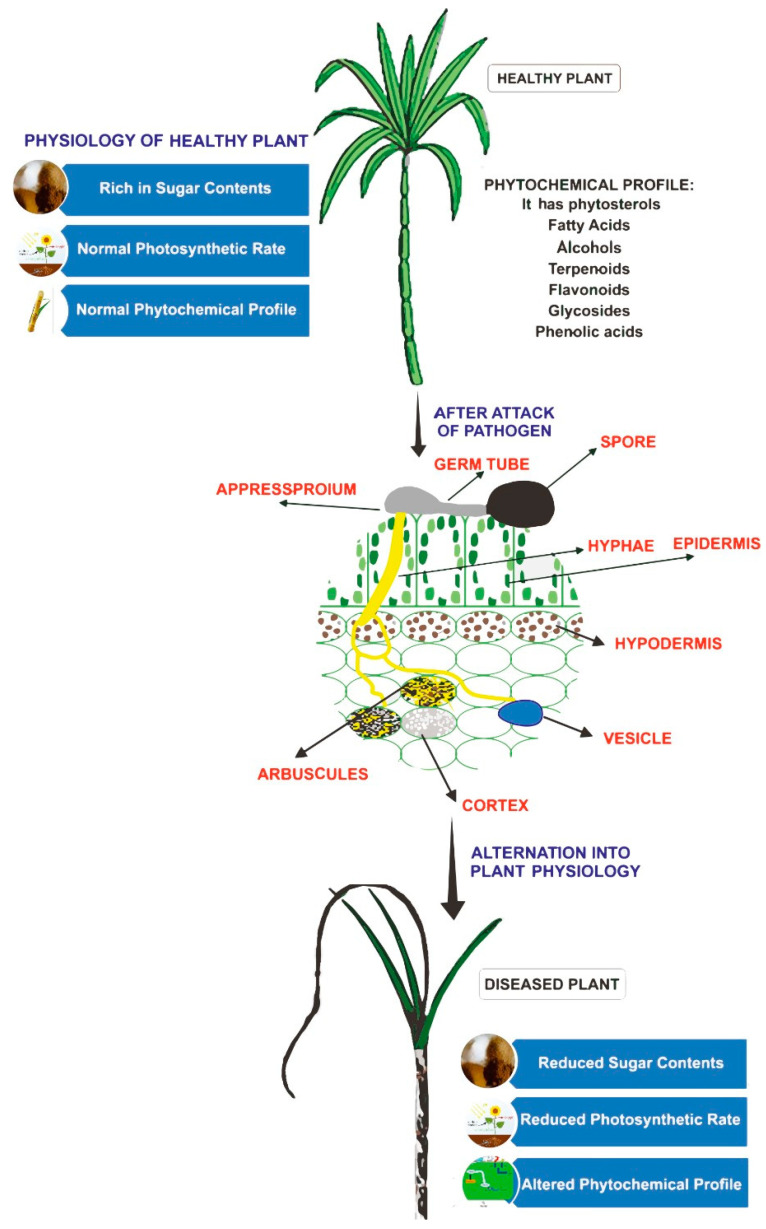
Effects of *Sporisorium scitamineum* infection on a normal sugarcane plant.

Sugarcane smut is caused by the dimorphic basidiomycetous fungus *S. scitamineum* (Sydow) M. Piepenbr., M. Stoll & Oberw. (Syn: *Ustilago scitamiea* H. & P. Sydow). It was first reported in Natal (South Africa) in 1877 [64]. It is one of the most prevalent diseases of sugarcane in almost all cane growing regions of the world except Papua New Guinea and Fiji [65,66,67]. Sugarcane is the C4 plant with a complex polyploid nature. A healthy plant has a normal photosynthetic rate and is rich in sugar content. However, once attacked by *S. scitamineum*, alternation into plant physiology occurs. The pathogen alters the photosynthetic rate and diseased plant has reduced sugar content. The pathogen also inhibits the expression of the defense-related genes (Figure 2). Whip smut is basically a disease of meristematic tissues, and the pathogen only propagates in young and actively growing plant tissues. Primary infection takes place either by soil-borne teliospores or planting infected setts, while secondary infection occurs through airborne fungal spores infecting standing healthy crop [40,68]. It enters the healthy sugarcane plant through lateral buds, in a very characteristic manner, i.e., by producing a typical black whip [67,69,70]. From one season to another, the whip smut usually perpetuates through propagative material and/or pathogen propagules present in the soil. The etiological agent may be present asymptomatically within the apparently disease-free setts [38]. Different developmental stages (teliospores, sporidia and mycelia) of *S. scitamineum* in vitro as well as inside the host are studied by protoplast-mediated transformation technique [71]. Green fluorescent protein (GFP) gene tagged *S. scitamineum* has efficiently revealed the whole phenomenon of pathogenesis from colonization to whip development in sugarcane plants [72].

Sugarcane smut is characterized by the emergence of a typical structure, called “smut whip”, the most identifiable diagnostic feature of infected plants [64]. The infected sugarcane plants are usually stunted in growth and produce thin slender canes, generally with broad-spaced nodes, having a whip-like sorus either at the top of affected stalks or on the side shoots of standing canes [65]. Generally, the infected sugarcane plants produce profuse tillers with the shoots being spindlier and erect with smaller narrow leaves [73,74]. The affected plants are severely stunted and yield losses may have a range of 12–75%. These are common in susceptible genotypes. However, a total crop failure may be possible, if susceptible cultivars are grown and climatic conditions are favorable for infection [17,40,65,75]. Temperature 25–30 °C and relative humidity 65–70% favor the disease development [76]. Compared to the plant crop, the losses are higher in ratoon crop [77,78,79,80,81,82]. Different workers from elsewhere reported varied losses in different cultivars and climatic conditions, such as 10–30% yield and 3–20% sugar losses [83], 68–80% yield and 32% in juice quality [84], 62% in yield [85] and 40–90% in sugar [86].

It is known that the practice of ratooning has also influenced smut intensity and resulted in yield losses. Ratooning of susceptible cultivars significantly increased smut incidences, causing tremendous yield losses of about 67% in the third ratooning [87]. Ratooning of a chewing type cultivar, highly susceptible to whip smut, should be avoided, otherwise it may result in a huge number of diseased stools and the complete yield loss [88]. In terms of yield, the chewing type sugarcane cultivar was suffering more with high inoculum load of *S. scitaminem* as compared to the industrial type sugar cultivar [15].

The host–pathogen interactions are studied by doing transcriptome profiling of *S. scitamineum* and sugarcane [89]. The proteomic studies revealed the remarkable variation in number and type expressed proteins in susceptible and resistant sugarcane cultivars [90]. Chorismate mutase, an effector of whip smut pathogen and several proteins in sugarcane are identified, which related with plant defense and other functions [91]. In *S. scitamineum,* SsSln1 histidine kinase receptor boosts as well as helps in operating the mating and virulence [92]. The transcriptome analysis also indicates that deficient mutant *SsΔMAT-1b* drops its normal mating and infection competency, which is expressed by bE/bW heterodimeric transcriptional factor [93]. It is demonstrated that in *S. scitamineum*, SsPEP1 effector not only regulates the pathogenesis and subsequent disease development as whip structures, it also operates the sexual mating and teliospores formation. Any disruption or deletion in its allele(s) can alter the whip formation and virulence capability [94]. During the interaction of a high virulent strain of *S. scitamineum* with a susceptible host, plant cell wall degrading enzymes (PCWDEs) such as chitinase-1 and laccase and secreted effector protein-coding (CSEPs) genes such as *SUC2*, *SRT1* and *CMU1* may be involved in regulating and enhancing the pathogen penetration and establishment in host tissues [95]. Recently, a number of genes belonging to the carotenoid cleavage oxygenase (*CCO*) and Ca^2+^/cation antiporter (*CaCA*) family in *Saccharum* spp. were identified, among which several gene members influence whip smut infection and other stresses [96,97]. 

## 3. Quantitative and Qualitative Parameters

The losses in yield and qualitative parameters are directly related to the smut severity, the more smut the more losses [98]. The smut development is directly responsible for reduction in sucrose as well as negatively affects purity and ultimately yield of the producible sugar [99,100]. The number of whips in a unit area also correlates with yield losses. In an experiment, production of 6265 whips/ha causes yield losses of 3.85 metric tons/ha [101]. Proportion of infected stalk or stools are also the scale of measuring yield losses. The study revealed that 1% stalk infection with smut disease corresponds to 0.46 metric tons/ha yield losses and 0.045 tons/ha sugar losses, while 1% stool infection resulted in 0.66 metric tons/ha yield losses and 0.06 tons/ha sugar losses [102]. In quantitative parameters, the most affected trait by smut was the number of healthy stalks or millable cane that was greatly reduced in susceptible cultivars. The increasing level of smut disease directly determined the reduction in cane yield per unit area [103].

The nature and extent of damage caused by whip smut disease mainly depend on the varieties under cultivation. The evaluation of 20 commercial varieties against whip smut disease revealed that the number of millable canes, plant height and cane girth were higher in resistant cultivars [104]. The *S. scitamineum* infection in susceptible cultivar caused the development of a very tiny stalk, having no commercial use, thus resulting in a considerable reduction in yield [105]. In some cases, especially in a highly susceptible cultivar, yield dropped to 40% in Australia. However, low susceptible varieties showed better tolerance to smut and performed well in terms of yield [106]. The reduction in yield parameters is directly correlated with the rate and amount of whip smut development in the fields. The field inoculation v/s un-inoculation of resistant, intermediate and susceptible cultivars revealed the sequential reduction in yield. The inoculation of *S. scitamineum* caused the yield losses of 20–33% in resistant or immune cultivars, 17% in intermediate/tolerant and 38% in susceptible varieties as compared to the uninoculated plots of the same cultivars [107]. Artificial infestation of two commercial cultivars with different inoculum level of *S. scitamineum* brought a significant reduction in all qualitative parameters except reducing sugars that enhanced in infected plots [108].

Pathogen infection in the planting setts had drastically reduced germination percentage and yield. Only 14 and 31% plant germination were recorded in naturally infected and artificially infested setts, respectively, as compared to the 40% germination in the healthy setts. Moreover, yield was reduced to 25 tonnes/hectare in artificially infested setts as compared to 43 tons in healthy setts [18]. A study conducted in Queensland, Australia predicts the maximum yield loss up to 62% in plots having very high disease of whip smut, while the average yield losses were about 26% [85]. The yield losses of more than 25% due to smut were reported from cane-growing areas of Queensland, Australia during 2009–2010. Farmers in these areas eradicated the severely infected standing crop to avoid the total losses [109].

Smut infection severely affects all quantitative as well as qualitative parameters in commercial cane cultivars (CoJ-64 and B.O-110). In these cultivars, plant height, cane girth, internodes, physical yield, juice quantity and quality were reduced due to *S. scitamineum* infection [20]. In Tanzania, the effect of *S. scitamineum* infection on seven cultivars was accessed and significant reduction in cane height, cane girth, yield and sucrose content of all cultivars was observed [110]. In Pakistan, the effect of *S. scitamineum* was evaluated on the qualitative and quantitative parameters, i.e., cane height, cane girth, brix, pol, purity and sucrose contents, of seven cultivars and there was a significant reduction in all parameters except commercial cane sugar content (CCS, expressed as a percentage) [76]. In Sudan, smut infection brought a significant reduction of 40% in cane weight, 5% in brix, 4% in sucrose content and 5% estimated recoverable sugar compared to the healthy crop. On the other hand, smut infection increased 7% fiber content in affected plants [111]. The inoculation of *S. scitamineum* increase the lignin contents of both resistant and susceptible cultivars by the activation of coniferyl alcohol dehydrogenase and sinapyl alcohol dehydrogenase [112].

## 4. Genetic Variability in *Sporisorium scitamineum*

In case of whip smut pathogen, *S. scitamineum,* the detection of diversity in earlier studies was mainly based on the specific cultivar’s response to pathogen infection and subsequent disease development. A report indicated that the first race of *S. scitamineum* designated as ‘Race A’ was first recorded in Hawaii (USA) in 1971 and ‘Race B’ in 1976. Variation in response of four varieties was the basis of race differentiation [113]. It also reported that during the late 1970s, both Races A and B were isolated from susceptible as well as resistant varieties in Hawaii [114]. In this context, in Taiwan, a study revealed race 3 of the smut pathogen, along with already confirmed races 1 and 2. In that study, the teleiospores are developed as a result of the mating of compatible sporidia of race 1 and 2 and subsequent inoculation on known highly susceptible cultivar. The differential set of cultivars produced symptoms differed with those of races 1 and 2 [115]. Based on the screening of 30 cultivars, the possibility of the presence of a new race other than races 1, 2 and 3 of the smut pathogen in Guangdong province, China was indicated [116,117]. All three races were already known to be present in neighboring Taiwan [116]. Seven major commercial varieties evaluated against whip smut disease under field conditions by artificial infestation with *S. scitamineum* at Guangxi bordering Taiwan and Guangdong (China) also revealed the possibility of presence of race 1, 2 and 3 [117].

Host responses under control conditions were also observed for race differentiation. A greenhouse study involving seven sugarcane cultivars and pathogen isolates collected from Argentina, Florida, Hawaii, Taiwan and Zimbabwe indicated the presence of six distinguished races [118]. Similarly, a series of consecutive glasshouse and field pathogenicity trials involving *S. scitamineum* isolates of two main sugarcane regions of Australia on the same sets of cultivars clearly indicated the variation between the isolates of these two regions [119].

Globally, efforts have been initiated to develop a set of standard host differentials, which include cultivars with a known response to certain races. In a broad-spectrum study, a set of 11 cultivars was planted in 10 countries to map the races of smut pathogen, which depicted the overall present variation to some extent but a high level of pathogen diversity was found in Taiwan only [120]. Similarly, a set of 11 cultivars was also used in Kenya to determine the prevailing races of *S. scitamineum.* Before planting, individual sets were inoculated with teliospores collected from different regions of Kenya separately. In terms of whip development, the tested cultivars behaved differently to various teliospores, indicating the presence of divergent *S. scitamineum* races in Kenya [82]. It is interesting that some investigators tried to differentiate the smut races on the basis of growth and colony characteristics of the *S. scitamineum.* Colony growth appearance was produced from the single teliospore or sporidia at different incubation temperatures varied with the isolates [121]. On the potato dextrose agar (PDA), race 1 at 26 °C produced creamy and white growth, while race 2 showed smooth and white growths. At 30–34 °C, race 1 produced creamy and yeast-like growth, whereas race 2 produced dark brown colonies with short monokaryotic hyphal growth. Similarly, a significant variation was detected in an Ethiopian population of smut pathogen based on the colony and growth characteristics, but the same isolates did not show any variation in the pathogenicity study [122].

With the passage of time, molecular tools became widely used for genetic diversity studies of phytopathogens. In China, to study the genetic diversity by the RAPD method in a smut pathogen population, 18 isolates of *S. scitamineum* were collected from six provinces. UPGMA cluster analysis divided the collected *S. scitamineum* population into six groups, and pathogen diversity was mainly geographically bound, but not originated from different cultivars [123,124]. Similarly, in Pakistan, 36 isolates collected from 12 different cane-growing areas of *S. scitamineum* were grouped into six distinguished clusters by the RAPD method [125]. The inter-simple sequence repeat (ISSR) and start codon targeted (SCoT) markers grouped Chinese *S. scitamineum* population into three distinguished clusters. The setts of 10 specific cultivars when inoculated with representative isolates of these three clusters produced differential disease reactions, thus confirming the presence of three distinguished races of the smut pathogen in China [126]. In another study, RAPD and ISSR analysis of Southern China population of the whip smut pathogen also revealed the presence of three races of *S. scitamineum* [127]. AFLP and telRFLP molecular markers were also used to evaluate the genetic diversity studies among the Brazilian and Argentine collection of *S. scitamineum,* which revealed the presence of two well-distinguished groups in the collected population [128]. In Philippines, a diversity study among 96 isolates of *S. scitamineum* collected from 17 different cane growing regions of the country was conducted at the molecular markers (10 microsatellite), pathogenicity (reaction of differential cultivars) and morphological level (teliospore size and shape). Cluster analysis as well as principal component analysis grouped all these populations into three distinct clusters. A pathogenicity study conducted on a set of five host differentials revealed seven groups in the Philippines’ smut population, whereas a morphological study failed to reveal any significant variation [129]. A similar trend was also observed during diversity studies of the Egyptian population of *S. scitamineum* by scanning electron microscopy (SEM) and using six random primers. SEM did not reveal any noticeable variation in the morphology of different teleiospore samples. However, molecular study indicated a significant diversity in the collected population of smut pathogens belonging to different Governorates [130].

It should be stressed here that some controversies also exist in the usefulness of the particular molecular and morphological techniques. After diversity study of 23 isolates of *S. scitamineum* belonging to six different provinces of China by ITS sequencing, it was concluded that ITS-based phylogenies failed to detect the genetic variation within the collected population of the smut pathogen [131]. In contrast, a study conducted in South Africa to determine the diversity among the *S. scitamineum* population of South Africa, Reunion Island, Hawaii and Guadeloupe by several methods revealed that only sequence data, especially ITS regions, showed a significant variation. Other methods, including a germination study, microscopy and morphological studies, RAPD and *bE* mating-type gene detections, showed no variation in the collected population [63].

In addition, studies also revealed that Asia, specifically Southeast Asia, is the center of *S. scitamineum* diversity. AFLP analysis of 38 worldwide collections of whip smut pathogen collected from 13 countries revealed the overall low diversity, but a significant level of diversity was observed in Southeast Asian populations of *S. scitamineum* [22]. DNA fingerprinting of the *S. scitamineum* population of Australia and Indonesia revealed no genetic variation in isolates. However, remarkable variation exists within the population of Taiwan, Thailand and Philippines. Moreover, isolates of these countries showed much more variation with the smut population compared to the rest of the world [132]. By using 17 microsatellites, a diversity study comprising 142 teliospores of *S. scitamineum* harvested from 77 smut whips, which were collected from 15 cane growing countries, revealed that a very low level diversity exists among African and American smut population as compared to the Asian population of *S. scitamineum,* which appeared as the major source of smut diversity [66]. Interestingly, whereas the molecular variation of *S. scitamineum* was associated with a geographic origin, there was no evidence of co-evolution between sugarcane and the pathogen [22].

## 5. Molecular-Based Detection of *Sporisorium scitamineum*

Pathogen may be detected even earlier than the symptom expression by using species-specific primers. Many workers successfully amplified and visualized *S. scitamineum* DNA present in the plant tissues by using appropriate primers [133]. Keeping in view the number of similarities in morphology, genetic makeup, life cycle and reproduction of *S. scitamineum* with other smut producing species such as *Ustilago maydis* and *U. hordei*, the DNA of sugarcane smut pathogen was amplified by using *U. maydis bE4* and *bE8* primers. The resulting PCR products showed 68–71% similarity with those of the *U. maydis bE* gene. Based on the above-mentioned sequence, *S. scitamineum bE* primers were synthesized, which successfully amplified the low quantity of whip smut DNA [27]. 

Irrespective of mating types, it accurately detects even the very minute pathogen propagules present in the plant, which was not detectable by microscopy [28]. The same whip smut-specific primers bE4 (5′-CGCTCTGGTTCATCAACG-3′) and bE8 (5′-TGCTGTCGATGGAAGGTGT-3′) successfully amplified the pathogen DNA at 459 bp both in resistant and susceptible cultivar, after 14 h of inoculation until one month of inoculation [29]. In another study, after 2 months of *S. scitamineum* inoculation, plant tops of sugarcane cultivars were harvested for DNA extraction and PCR amplification by using S1 and S2 intergenic spacer region primers (3′-GCAGCCGATAATCTACCAA-5′ and 5′-CCAGCTTCTTGCTCATCCTC-3′) [27,28,29]. On gel electrophoresis, the susceptible and highly susceptible clones revealed the smut-specific amplicon of 450 bp, while the same was not detected in resistant clones [134]. More recently, a loop-mediated isothermal amplification (LAMP) protocol was reported for the early detection of whip smut pathogen in the plant tissues. This technique is more robust, sensitive and accurate as well as more economical than PCR based *S. scitamineum* detecting assays [30]. By using molecular diagnostic assay, pathogens may be detected even earlier than the symptoms expression [135]. However, to measure the effects of disease on plant growth and yield, conventional screening methods are still desirable. An optimized inoculation method is a prerequisite for a reliable and successful screening program [34].

## 6. Smut-Resistant Germplasm Screening

The whip smut disease can effectively be managed by broad scale selection and planting of resistant cultivars as the most reliable, economic and effective control strategy [136,137,138]. The development of smut-resistant varieties is the focus of sugarcane breeding programs worldwide [139]. Studies have revealed that polygenic resistance towards this disease is present in sugarcane cultivars, as suffered plants show different types of reactions [61]. The incidence was quite high in blocks of susceptible cultivar and gradually declined in moderately resistant or resistant cultivars [103]. The high level of susceptibility to whip smut forced to stop the cultivation of superior cultivars [45,46,47]. 

Screening of widely grown commercial varieties along with potential lines is an old and ongoing process. For accessing varietal response to whip smut, different disease rating scales were proposed from time to time. A five-category disease rating scale for accessing the host response against whip smut was proposed in 1969 [140]. Afterward, a 1–9 disease rating scale, which has some ambiguities, was described [141], where 1: 0–3% (HR), 2: 4–6% (R), 3: 7–9% (R), 4: 10–12% (R), 5: 13–25% (MS), 6: 26–35% (S), 7: 36–50% (HS), 8: 51–65% (HS) and 9: 66–100% (HS). Later, a four-level disease accessing system was suggested, where R: 0–5%, MR: 5.1–15%, MS: 15.1–30% and S: above 30% [142]. A more comprehensive 0–9 scale was described in 1980, which is still widely used by the research workers, where 0: no disease, 1: 0.1–2.5%, 2: 2.6–5.5%, 3: 5.6–7.5%, 4: 7.6–12.5%, 5: 12.6–15.5%, 6: 15.6–18.0%, 7: 18.1–22.5%, 8: 22.6–25.5% and 9: 25.6–100% [61].

Resistance screening is a very old practice to combat whip smut disease. In India, among 11 cultivars, artificially infested before planting, four appeared susceptible, three moderately susceptible and three moderately resistant [143]. In another study carried out in India, only 7 out of 20 cultivars were resistant against whip smut [104]. A report indicates the field evaluation of 84 cultivars in Brazil artificially inoculated with the smut pathogen [144]. Until 1974, the whip smut disease spread widely in Hawaii, USA. About 70% of commercial varieties showed various levels of susceptibility to whip smut [145]. Therefore, large-scale varietal evaluation programs were initiated to find out resistant germplasm against this devastating disease [145,146]. In a comprehensive sugarcane breeding program of Australia, 1705 and 481 accessions were planted in neighboring Indonesia and the ORIA region of Western Australia in 1998, which revealed that most screened cultivars (69%) are susceptible to smut. Afterwards, 1007 and 1600 cultivars were screened in Queensland during 2006 and 2007, respectively [147].

A field trial conducted at Guangxi province, China revealed that among seven commercial cane varieties, which were artificially inoculated with smut pathogen, three appeared as moderately resistant, two moderately susceptible and two were susceptible to whip smut infection [117]. In another study, among 30 cultivars, which were inoculated with pathogen prior to sowing screened under field conditions, nine were highly resistant, three resistant, three moderately resistant, three moderately susceptible, nine susceptible and three highly susceptible [116]. Out of 34 sugarcane cultivars planted under field conditions in Guangdong province (China) after inoculation with *S. scitamineum*, five showed highly resistant reaction, four resistant, two moderately resistant, eight moderately susceptible, seven susceptible and nine were highly susceptible to whip smut [138]. In Bangladesh, 43 cultivars screened under field conditions after artificial inoculation with smut pathogen showed that five were highly susceptible, 16 susceptible, seven moderately susceptible, three moderately resistant and 12 resistant to whip smut disease [148]. Among 32 cultivars obtained from the French Agricultural Research Centre for International Development (CIRAD) and screened under Ethiopian conditions, three were very highly susceptible, two susceptible, one intermediate, nine moderately resistant, seven resistant, three highly resistant and seven very highly resistant [149]. Wild relatives and hybrids of cultivated canes also appeared good source of resistance against whip smut. A study conducted in Sri Lanka revealed that out of 455 entries artificially infested with *S. scitamineum*, 124 were free from infection, including 86 hybrids, 16 cultivars of *Erianthus arundinaceus* and 16 of *Saccharum spontaneum* [150]

For finding a resistant germplasm, various strategies have been utilized, including early testing of plants for smut resistance using pathogen proliferation and changes in physiological and biochemical indices, induced mutagenesis, tissue culturing, backcrossing with allied species and the evaluation of exotic varieties and breeding materials [135,151]. Plants yielded from somaclonal variation of commercial varieties also showed resistance against whip smut disease [134]. Some of the clones that were produced as a result of back crossing of commercial sugarcane varieties having desirable yield characters with sweet cane (*Erianthus arundinaceus*) appeared a good source of resistance against whip smut disease [138]. The induced mutation would be one of the effective strategies to evolve resistant cultivars, as a study revealed that out of 41 mutants, 11 appeared highly resistant when buds were exposed to *S. scitamineum* before planting [152]. Ethyl methyl sulfonate (EMS)-induced mutants of commercial cultivars were resistant to smut and performed well in qualitative parameters [151]. Discovering *S. scitamineum* effectors and their plant target genes reveals the events of the host–pathogen interaction at a molecular level. The differentially expression profile of genes in susceptible and resistant sugarcane cultivars were also reported [153,154]. In another attempt, 13 out of 861 differentially expressed genes were identified conferring resistance in cv. CP74-2005 against *S*. *scitamineum* [155]. Moreover, the levels of genes such as sugarcane *dirigent16 gene* (*SofDIR16*) and sugarcane *cinnamyl* alcohol dehydrogenase gene (*SofCAD*) were also observed to vary in resistant and susceptible cultivars even prior to infection. It also appeared that *SofDIR16* stimulates the lignan formation in whip smut-resistant cultivars [156]. The expression of disease resistance-related genes including *PR10*, *HCT1* and *ScChi* was regulated in resistant cultivars immediately after infection and gradually decreased with passage of time. However, in susceptible cultivars, their expression was enhanced with the passage of time [157].

## 7. Chemical Control

Fungicides provide a preventive and curative defense against whip smut disease [43]. Testing of fungicides against this disease is an ongoing practice (Table 1). In the early 1970s, fungicidal dip of setts before planting with Agallol and Dithane Z-78 appeared highly effective among seven tested fungicides against whip smut disease [158]. Dipping the setts in Aretan (Methoxy ethyl mercury chloride) solution before planting also gave highly effective season-long control of whip smut disease [159]. Another study showed that dipping the setts prior to planting in the fungicidal solution of Captafol 60 (Captafol) appeared highly effective in providing maximum yield [160].

The duration of the setts’ dip in fungicidal solution is influenced on the efficacy of the fungicides as well as the disease development. Dipping the setts in Triademifon (0.1%) or Propiconazole (0.1%) for four hours completely eradicates the smut infection, while some incidence was developed when setts were dipped for two hours in the same fungicides [162]. In many cases, the effectiveness of the fungicides becomes enhanced when used with hot water instead of the ambient one. Hot water fungicidal dip of Triadimefon appeared highly effective against whip smut as well as stunting disease as either sett was inoculated with smut pathogen prior to planting or planted in the soil artificially infested with the teliospores of *S. scitamineum* [163]. Even with comparison with biocontrol agents such as *Pseudomonas fluorescens* and *Trichoderma viride* + *P. fluorescens* + *Bacillus subtilis,* Triadimefon appeared highly effective against whip smut. However, the efficacy of fungicides may vary with the cultivars [164,165,166].

The new chemistry fungicides, such as Cyproconazole, Propiconazole and Azoxystrobin, also appeared effective [168]. For setts artificially infested with *S. scitamineum* dipped in a fungicidal solution containing different individual fungicides or their combinations, the most effective treatments were Pyroquilon followed by Carbendazim + Maneb and Metalaxyl + Carboxin + Furathiocarb [137]. Sett treatment with Carbendazim alone produced a significantly higher germination, cane yield and CCS as compared to the Carbendazim + gibberellic acid and control (setts without any treatment) [170]. In highly susceptible cultivar, spray of Flutriafol on setts at planting provides highly satisfactory disease control as well as brought remarkably increase in yield as compared to the inoculated plots of the same cultivar [167]. It was also reported that Flutriafol has more penetration into plant tissues than other competitive fungicides. By eradicating the already-established *S. scitamineum* infection, it provides the effective protection to highly susceptible cultivars for a longer period against smut [171]. In ratoon crop, which tends to be more affected with whip smut disease, the use of Spectrum (Azoxystrobin + Tebuconazole) as sett treatment and the foliar application of Tebuconazole at the time of ratoon initiation and one month of ratooning can effectively minimize the disease intensity [169].

In most cases, the application of fungicides led to a significant increase in the yield and quality of sugarcane [166]. The new fungicide Flutrifol not only provided highly satisfactory whip smut control, but also brought a remarkable increase of up to 203% in yield [167]. Triadimefon appeared as highly effective fungicide, followed by Propiconazole and Kresoxinmethyl + Hexaconazole in reducing the smut incidence and producing a maximum cane yield. The applications of Triadimefon produced a remarkable maximum yield of 153 t/ha as compared to 113 t/ha recorded in control (no fungicide) [164].

## 8. Biological Control

Although the use of fungal and bacterial antagonists as well as botanicals against a number of infectious plant diseases has gaining more attention since long, so far, very few studies have been conducted on the non-chemical control of sugarcane smut. Some botanicals supported and enhanced the spores’ germination and vegetative growth of *S. scitamineum*, while others caused negative effects on mycelial growth and teliospore germination [172,173]. Under in vitro conditions, the 10% leaf extracts of *Calendula officinalis*, *Solanum nigrum* and *Eclipta alba* brought about 90% inhibition in teliospore germination of *S. scitamineum*, while another 20 plant extracts had positive effects on the spores’ germination. Sett treatment with extract of *S. nigrum* also suppressed smut development under field conditions [173]. Pre-planting treatment of artificially infested setts with the essential oil of lemon grass *Cymbopogon citratus* provides the effective control of whip smut as well as enhanced quantitative traits [174]. Among bacterial antagonistic, a specific strain of *P. guariconensis* caused disruption in the usual sexual mating of *S. scitamineum* sporida, which could lead to the inhibition in eventual hyphae development and pathogen penetration in the host tissues [175]. Further investigation revealed that 4-hydroxybenzaldehyde and indole-3-carbaldehyde are two of the active compounds isolated from this specific strain. Both compounds either controlled the vegetative growth and/or obstructed the mating process of *S. scitamineum*. Additionally, indole-3-carbaldehyde application remarkably reduced the intensity of corn smut in treated plants [176]. Whole genome sequence analysis of B18 strain of *P. aeruginosa* indicated the presence of *hcnABC*, *phzA_B*, *phzDEFGMS* and *pchA* genes, which regulated its antagonistic function against *S. scitamineum*. This specific strain of *P. aeruginosa* was very effective in enhancing plant growth as well boosting the plant defense mechanism against whip smut infection [177].

Endophytic bacteria, especially certain *Bacillus* spp., also showed promising results against sugarcane smut. However, comprehensive screening is the prerequisite to find out effective strains, as out of 119 strains, only eight showed antagonistic potential under in vitro conditions, while four remarkably reduced smut development in greenhouse conditions and only two i.e., *Bacillus pumilus* and *B. axarquiensis*, performed well under field conditions. These two species not only brought significant reduction in whip smut development, but also enhanced quantitative and qualitative parameters [178]. Similarly, compounds isolated from specific strain of *B. siamensis* showed antagonistic characteristics against whip smut pathogen [179]. One specific strain of bacterial pathogen *Burkholderia gladioli*, which was isolated from sugarcane leaves, also showed biocontrol potential against whip smut in laboratory and field experiments [180].

A number of fungal antagonists, especially the members of *Trichoderma*, has also been evaluated against *S. scitamineum* [181]. Some saprophytic fungi, such as *Fusarium moniliforme* var. *subglutinans*, *Aspergillus flavus*, *A. niger* and *Penicillium* sp., naturally colonized the smut whip in fields, especially during the rainy season; thus, this reduced the risks of secondary infection [182]. The cultural filtrates of these fungi, especially *F. moniliforme*, inhibited the teliospore germination [183]. Mycophenolic acid, an antibiotic compound yielded from some *Penicillium* spp., is also known to adversely affect the development of dikaryotic hyphae of smut pathogen [184]. The cultural filtrates of *T. viride* considerably reduced teliospore germination, smut incidence and significantly enhanced sett germination and yield [173]. Other studies also revealed the strong in vitro inhibitory ability of *T. harzianum* and *T. viride* against *S. scitamineum.* They increased the sett germination and checked the smut infection in treated setts [161,185]. Beside disease control, Trichoderma species also act as plant growth regulators or biofertilizers. The application of metabolites, spore suspension and *Trichoderma* multiplied culture of *T. harzianum* and *T. viride* caused a profound increase in plant germination as well as yield parameters and CCS. However, *T. viride* performed better than *T. harzianum* in all modes of applications. Moreover, use of metabolites appeared more effective than spore suspension or multiplied cultures [186]. Recently, it was found that a sugarcane smut fungus effector can mimic the host plant elicitor peptide to compete its perception and, thus, suppress the receptor-activated immunity, which may give us a new insight on biocontrol [89].

## 9. Conclusions

As whip smut has the potential to cause substantial losses in susceptible cultivars, the varieties under cultivation should be replaced with resistant ones that have desirable agronomic characters. Regular monitoring, roughing and destruction of smut whip will help to reduce the inoculum. The ongoing and upcoming varietal development as well as cultivar screening programs should consider the presence of multiple races of smut pathogen in this region. There is a high need to study the rate and mechanism of the physiological specialization in smut pathogen as well as to confirm the presence of multiple races of *S. scitamineum* by recent molecular tools including race specific arrays. The changing diversity and virulence in *S. scitamineum* should also be monitored regularly in different ecological zones. Breeding and varietal screening programs should adopt molecular-based detection techniques for early detection of smut infection in germplasm. The effectiveness of sett pelleting as well as wide scale field-testing of antagonistic microbes such as *Trichoderma*, *Bacillus* and *Pseudomonas* should also be considered. Taken together, this review presents the current knowledge on various aspects of sugarcane smut and the disease management strategies, and future research directions are also proposed.

## 10. Future Prospects

Research efforts should be directed to cutting-edge research areas because of the diverse and complex nature of smut pathogens, including its economic significance. There is a need to directly focus on pathotyping and disease forecasting models. It is generally recognized that various biocontrol agents do not have the potential to thrive in newly introduced habitats, which is why it is also vital to introduce new antagonistic microbes. The nanotechnological approach may provide novel insights for us to develop a potential antifungal agent to combat the challenge caused by sugarcane smut disease.

## Figures and Tables

**Table 1:** Fungicides effective against whip smut disease.

Common Name	Trade Name	Chemical Group/Class	References
Methoxy ethyl mercury Chloride	Agallol, Aretan, Emisan, Ceresan wet	Organomercurials	[158,159,161]
Zineb	Dithane Z-78, Aphytora, Amitan, Tiezene	Dithiocarbamates	[158]
Captafol	Captafol 60, Foltaf, Difolaton, Difosan, Captaspor, Foleid	Heterocyclic Nitrogen Compounds	[160]
Triadimefon	Bayleton, Amiral	Triazole	[11,39,42,162,163,164,165,166,167]
Propiconazole	Tilt, Bumper, Propi Max EC, Heritage	Triazole	[11,42,161,162,164,166,167,168,169]
Cyproconazole	Alto	Azoles	[168]
Azoxystrobin	Amistar, Dynasty, Quadris	strobilurins	[168]
Pyroquilon	Coratop, Fongoren, Lilolidone	Hydroquinolones	[137]
Carbendazim	Carbendazim, Carbendazole, Bavistin, Mecarzole, Bavistan	Benzimidazoles	[42,170]
Flutriafol	Flutriafol, Sinker, Impact, Vincit, Rhyme, Topguard	Triazole	[167]
Triademinol	Bayfidan	Triazole	[11]
Cyprodinil	Vangard	Anilinopyrimidine	[39]
Azoxystrobin + Tebuconazole	Spectrum	Strobulirin & Triazole	[169]
Trifloxystrobin + Tebuconazole	Nativo, Patriot, Gezeko	Strobulirin & Triazole	[169]
Carbendazim + Maneb	Delsen M	Benzimidazoles & Dithiocarbamates	[137]
Metalaxyl + Carboxin + Furathiocarb	Apron Plus	Acylalanine, Oxathiin (anilides) & Coumarans	[137]
